# A Novel Mitochondria‐Associated Programmed Cell Death–Related Prognostic Model and Validation of Oncogene INHBB in Colorectal Cancer

**DOI:** 10.1155/ijog/8691810

**Published:** 2025-11-26

**Authors:** Yanqing Sun, Chun Gao, Dong Jia, Qiang Ma, Wei Wang

**Affiliations:** ^1^ The First Clinical Medical College, Gansu University of Chinese Medicine, Lanzhou, Gansu, China, gszy.edu.cn; ^2^ Department of Gastroenterology, 940th Hospital of Joint Support Force, Lanzhou, Gansu, China

**Keywords:** colorectal cancer, immune infiltration, mitochondrial, programmed cell death

## Abstract

**Objective:**

The objective of this study is to explore mitochondria‐associated programmed cell death (mtPCD)–related key biomarkers for patients with colorectal cancer (CRC).

**Methods:**

CRC‐related datasets were obtained from the GEO and TCGA databases, and mitochondria‐related genes (MitoRGs) and PCD‐related genes (PCDRGs) were acquired from the MitoCarta database or pertinent literature. After differentially expressed gene (DEG) screening, the DEmtPCD coexpressed genes were identified. Then, consensus clustering analysis was conducted, followed by GSEA and immune infiltration analysis. In addition, a prognostic model was constructed, and then, immune infiltration analysis, GSEA, and drug sensitivity analysis were carried out. The qRT‐PCR and western blot were employed to determine the expression of key genes. Finally, a loss‐of‐function experiment was applied to investigate the influence of INHBB on CRC in vitro.

**Results:**

A total of 2118 DEGs were screened, and then, 64 DEmtPCD coexpressed genes were obtained. Subsequently, two clusters, including C1 and C2, were identified, and patients in the C1 group had better survival outcomes. In addition, a prognostic model was constructed based on four key genes, namely, ACSL6, INHBB, GPR15, and SRPX. Also, the area under the curves (AUCs) for overall survival (OS) at 1, 3, and 5 years were 0.667, 0.665, and 0.603 in the TCGA set, separately, and 0.759, 0.766, and 0.662, separately, in the GSE17537 dataset. The total fraction of nine immune cells showed a significant difference between the low‐ (L) and high (H)‐risk groups, such as neutrophils, activated NK cells, and activated dendritic cells. Also, there was a significant difference in TIDE scores between the H‐ and L‐risk groups, and APC was the most significantly mutated gene in both the H‐ and L‐risk groups. IC_50_ value of some chemotherapeutic agents showed a significant difference between the L‐ and H‐risk groups, containing AZD1332‐1463, IGF1R‐3801‐1738, and XAV939‐1268. The expression levels of ACSL6, GPR15, and INHBB were significantly elevated in CRC cells compared to those in NCM460 cells, while SRPX significantly decreased. Notably, downregulation of INHBB effectively alleviated the tumorigenesis of SW620 cells.

**Conclusion:**

A prognostic model was constructed based on four key mtPCD‐associated genes, namely, ACSL6, INHBB, GPR15, and SRPX. INHBB was upregulated in CRC, and the alleviation of INHBB suppressed the proliferation and migration of CRC cells.

## 1. Introduction

Colorectal cancer (CRC) is the third most common cancer worldwide and the second leading cause of cancer‐associated deaths [1]. In the past two decades, the age of onset of CRC has been decreasing, and the proportion of patients with advanced CRC has gradually elevated in the past decade, with an unsatisfactory overall survival rate [2–4]. Therefore, the search for new biomarkers and effective treatment strategies is of great importance for the diagnosis and treatment of CRC.

Mitochondria are essential organelles in the human body, primarily responsible for producing ATP [5]. In addition, mitochondria exert a significant function in the production of reactive oxygen species (ROS), regulation of cellular signaling, and biosynthetic metabolism [6]. Numerous studies have shown that mitochondrial functions are closely linked to cancer progression. For instance, mitochondrial dysfunction is a prominent feature of cancer and has been associated with tumor metastasis and drug resistance [7]. The structure and function of mitochondria in tumor cells are obviously changed when compared with normal cells, such as metabolic activity alteration, ROS level elevation, and mitochondrial DNA mutation [8, 9]. Moreover, these mitochondrial alterations also exacerbate the progression of CRC [10].

Programmed cell death (PCD), a process that is mediated by a variety of ordered molecular events, exerts a significant function in health and disease [11, 12]. It is reported that PCD subroutines are a key feature of tumorigenesis [13]. Numerous forms of PCD, including apoptosis, autophagy, ferroptosis, pyroptosis, necroptosis, cuproptosis, NETosis, anoikis, and disulfidptosis, have been identified and are observed to exert a significant function in the development of CRC [14–16]. Furthermore, accumulating evidence emphasized the intricate interplay between mitochondrial dynamics and cell death pathways [17]. Mitochondria are the convergence point of multiple cell death induction pathways, which trigger various mechanisms of apoptotic and nonapoptotic PCD [18]. Li et al. found that METTL17 coordinates ferroptosis and tumorigenesis by regulating mitochondrial translation in CRC [19]. In addition, ROS levels in tumor cells with mitochondrial genetic abnormalities are significantly higher than in normal cells, and high levels of ROS can mediate cell death [20]. It is reported that the accumulation of intracellular ROS can induce mitochondria‐mediated apoptosis and autophagy initiation in CRC cells [21]. However, the biomarkers and mechanisms associated with mtPCD in CRC remain unclear.

**Figure 1:**
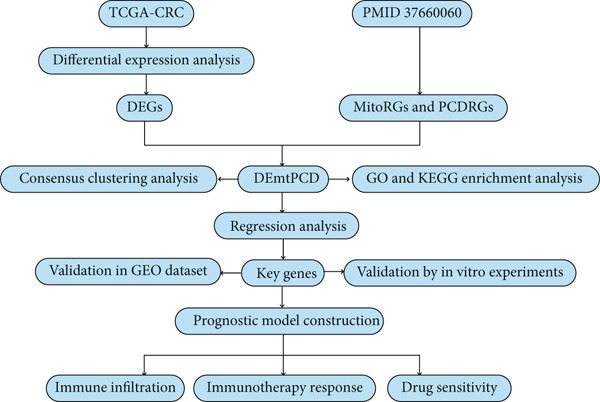
The graphic depicts the study′s process.

In this study, the mtPCD‐related key biomarkers and prognostic model for patients with CRC were explored, followed by validation in vitro. Figure 1 illustrates the study workflow. This study provides direction for the search for new CRC biomarkers and treatment options.

## 2. Methods

### 2.1. Data Source

The TCGA‐CRC (TCGA‐COAD&TCGA‐READ) dataset was acquired from the UCSC database (https://xenabrowser.net/datapages/), including 635 CRC samples and 51 normal samples, which were utilized as the training set. Besides, the GEO database was searched to obtain the datasets of the GSE17537 and GSE87211, serving as the validation sets. The GSE17537 dataset contained 55 CRC samples, and the GSE87211 dataset comprised 203 CRC samples and 160 normal samples. Furthermore, a total of 1136 mitochondria‐related genes (MitoRGs) were acquired from the MitoCarta3.0 database [22], and 1548 PCD‐related genes (PCDRGs) were searched by evaluating the pertinent literature [23]. Subsequently, the ssGSEA was carried out to compare the MitoRG and PCDRG scores between the CRC and normal groups using the “ssgsea” function in the GSVA package. The “DESeq2” package [24] (v 1.42) was employed to identify the differentially expressed genes (DEGs). The Benjamini–Hochberg (BH) method was applied to correct the *p* value, and *p*.adj < 0.05 and |log2FC| > 2 were utilized as the screening thresholds. To obtain the differentially expressed (DE) MitoRGs and PCDRGs, the obtained DEGs were intersected with the 1136 MitoRGs and 1548 PCDRGs, separately. Then, the Spearman correlation analysis was employed on the obtained DEMitoRGs and DEPCDRGs, and the genes with the cutoff value of *r* > 0.5 and *p* < 0.01 were considered as the DEmtPCD coexpressed genes. In addition, the enrichment analysis was carried out by “clusterProfiler” (v 4.4.4) with the threshold of *p*.adj < 0.05.

### 2.2. Consensus Clustering Analysis

“ConsensusClusterPlus” package (v 1.66.0) [25] was utilized to conduct consensus clustering analysis on the DEmtPCD coexpressed genes, and the CRC samples were divided into different subtypes, followed by DEGs screened between different subtypes with the cutoff value of *p*.adj < 0.05 and |log2FC| > 1. Then, the GSVA was employed to identify DE HALLMARK pathways between different subtypes with the cutoff value of FDR < 0.05 and |*t*| > 2.

### 2.3. Development of Prognostic Model

Univariate Cox regression analysis was conducted on the DEmtPCD coexpressed genes by the “survival” package [26] (v 3.5‐8), followed by LASSO regression analysis conducted by “glmnet” [27] (v 4.1‐8). Then, multivariate Cox regression analysis and the “step” function were employed to identify the key genes for prognostic model construction. The risk score was calculated with the formula: Riskscore = *h*0(*t*)∗exp(*β*1*X*1 + *β*2*X*2+⋯+*β* *n* *X* *n*) (*β* refers to the regression coefficient, *h*0 indicates the baseline hazard function, and *X* suggests the expression value of the gene). The “surv cutpoint” function in the “survminer” package [28] (v 0.4.9) was utilized to find the optimal threshold, and then, the samples were separated into the high‐ (H) and low (L)‐risk groups. The predicted performance of the prognostic model was confirmed by the Kaplan–Meier (KM) and ROC curves in the GSE17537 dataset. Also, the GSE87211 dataset was utilized to validate the expression of key genes.

### 2.4. Immune Infiltration Analysis

The “CIBERSORT” algorithm [29] was used to analyze the immune infiltration of the samples, and the Wilcoxon test was employed to compare the difference in immune cell infiltration between different subtypes or risk groups.

### 2.5. Drug Sensitivity Analysis

Based on the GDSC2 database (https://www.cancerrxgene.org/), the “oncoPredict” package [30] (v 0.2) was employed to analyze the association between risk score and drug sensitivity. The GSCALite database (https://guolab.wchscu.cn/GSCA/#/) was utilized to assess the association between the expression of key genes and the sensitivity of common drugs.

### 2.6. Cell Culture and Transfection

Normal colonic epithelial cell NCM460 (#SNL‐519, Sunncell) and CRC cell SW620 (#CL‐0225, Procell) were cultivated in DMEM medium (#C11885500BT, Gibco) supplemented with 10% FBS (#34080619, Ephraim) and 1% penicillin/streptomycin (#SV30010, Hyclone) at 37°C with 5% CO_2_. CRC cells HCT116 (#CL‐0096, Procell) and HT29 (#CL‐0118, Procell) were grown in RPMI‐1640 medium (#8120348, Hyclone). For transfection, the sh‐INHBB sequences were acquired from the DSIRI website. INHBB shRNA, sense: 5 ^′^‐CCATAGACTTGCTGTTAAA‐3 ^′^; antisense: 5 ^′^‐TTTAACAGCAAGTCTATGG‐3 ^′^. The SW620 cells were maintained in a six‐well plate, with 2 × 10^5^ cells/ well, and maintained until the cell density reached 70%–90%. Then, the SW620 cells were transfected with lentivirus containing either sh‐INHBB or its control (sh‐NC) for a duration of 24 h. To determine cell viability, a commercial CCK‐8 kit (#C0037, Beyotime) was applied.

### 2.7. Colony Formation Assay

SW620 cells (200 cells/well) were maintained in the six‐well plate and kept in a 5% CO_2_ incubator for 14 days. When visible clones appeared, the cells were fixed with 1 mL of absolute methanol (#80080418, Sinopharm), and the cells were stained with crystal violet (#C0121‐100 mL, Beyotime). Finally, the results were observed after removing excess dye.

### 2.8. Scratch Wound Healing Assay

The migration ability of SW620 cells at 0 and 48 h was assessed by the scratch wound healing assay. In short, the lines were drawn on the back of the six‐well plates with a marker pen, and SW620 cells were grown in the six‐well plates. The next day, a pipette tip was employed to create a “scratch” of the cell monolayer that was perpendicular to the back lines, followed by the implementation of serum‐free medium. Finally, the results were photographed at 0 and 48 h.

### 2.9. Western Blot

Total protein was acquired after lysis, and then, proteins were uncoupled by SDS‐PAGE and transferred onto PVDF membranes (#FFP24, Beyotime). After blocking, the membranes were probed with primary antibodies against ACSL6 (1:5000; #DF4142, Affinity), GPR15 (1:1000; #DF2727, Affinity), INHBB (1:1000; #AF5170, Affinity), SRPX (1:5000; #DF6349, Affinity), and GAPDH (1:10000; #ab181602, Abcam) at 4°C overnight incubation with secondary antibody goat antirabbit IgG (1:2000, #ab6721, Abcam) for 60 min. The results were developed by ECL (#P1000, Applygen).

### 2.10. qRT‐PCR

The cells were lysed with TRIzol reagent (#15596026, Ambion), then 5× FastKing RT SuperMix (#KR118, TIANGEN) was employed for cDNA synthesis, and then, qRT‐PCR was carried out by SYBR GREEN Realtime PCR Master Mix (#QPK‐201, Toyobo). Primer sequences contained the following: human ACSL6 (F) 5 ^′^‐CAGGAGCCTGCTGGAAAGAG‐3 ^′^ and (R) 5 ^′^‐TCGTCCTGTCTCGGAAAGA‐3 ^′^, human GPR15 (F) 5 ^′^‐TTGCATTTCAAACCCGGCAG‐3 ^′^ and (R) 5 ^′^‐CCGTCCTCCACAGTCCTAGA‐3 ^′^, human INHBB (F) 5 ^′^‐ATCTGTGCCCTTCATTGGGG‐3 ^′^ and (R) 5 ^′^‐TCAGATGGCCTCACCGTCTA‐3 ^′^, human SRPX (F) 5 ^′^‐CTTCCCAGGATCGGGAGACT‐3 ^′^ and (R) 5 ^′^‐TTGTTGGGTTCTGCAATGCG‐3 ^′^, and human GAPDH (F) 5 ^′^‐AGCTTCAGCCCCAGGAAATC‐3 ^′^ and (R) 5 ^′^‐GACATACTGCTGGGCCAGTT‐3 ^′^. GAPDH was applied as the internal reference.

### 2.11. Statistical Analysis

Data (means ± SD) were analyzed utilizing GraphPad 7.0. Differences between groups were explored to evaluate the expression and function of key genes by one‐way analysis of variance (ANOVA). Statistical significance was deemed when *p* < 0.05.

## 3. Results

### 3.1. Acquisition of 64 DEmtPCD Coexpressed Genes

A total of 2118 DEGs were screened between the CRC and normal groups, comprising 1074 upregulated and 1044 downregulated DEGs (Figure 2a,b; Table S1). In addition, MitoRG (*p* = 0.027) and PCDRG (*p* < 0.01) scores showed a significant difference between the CRC and normal groups, respectively (Figure 2c). Furthermore, the obtained DEGs were intersected with the 1136 MitoRGs and 1548 PCDRGs, separately, and 28 DEMitoRGs and 111 DEPCDRGs were acquired by taking the intersection set, respectively (Figure 2d). After Spearman′s correlation analysis, a total of 64 DEmtPCD coexpressed genes were obtained (Figure 2e). Besides, enrichment analysis was performed on the 64 DEmtPCD coexpressed genes (Figure 2f,g), and these genes were mainly enriched in the PPAR signaling pathway, the calcium signaling pathway, and the regulation of actin cytoskeleton pathways.

Figure 2Acquisition of 64 DEmtPCD coexpressed genes. (a) Volcano plot of DEGs obtained from the CRC and normal groups. (b) Heatmap of the top 50 DEGs obtained from the CRC and normal groups. (c) Difference in MitoRG and PCDRG scores between the CRC and normal groups. (d) Acquisition of DEMitoRGs and DEPCDRGs. (e) Spearman′s correlation analysis between DEMitoRGs and DEPCDRGs. (f) GO functions enriched by DEmtPCD coexpressed genes. (g) KEGG pathways enriched by DEmtPCD coexpressed genes.

**Figure:**
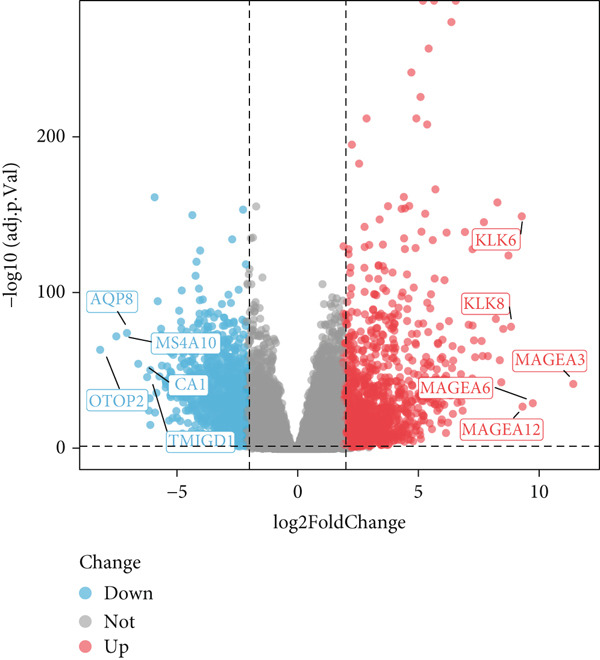
(a)

**Figure:**
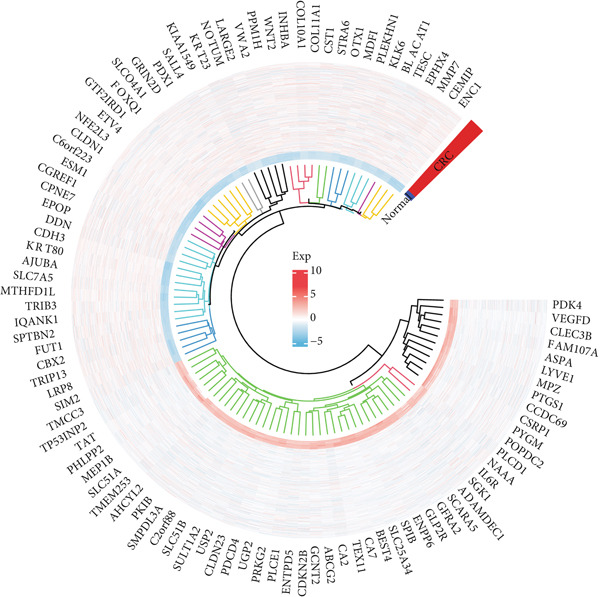
(b)

**Figure:**
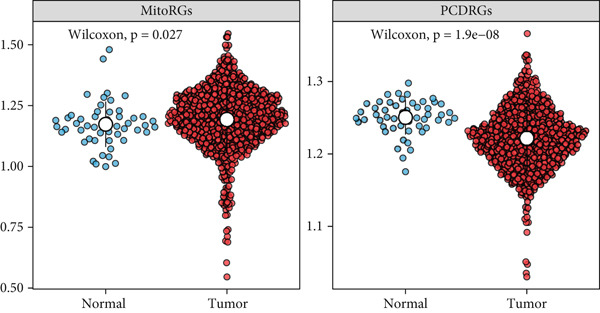
(c)

**Figure:**
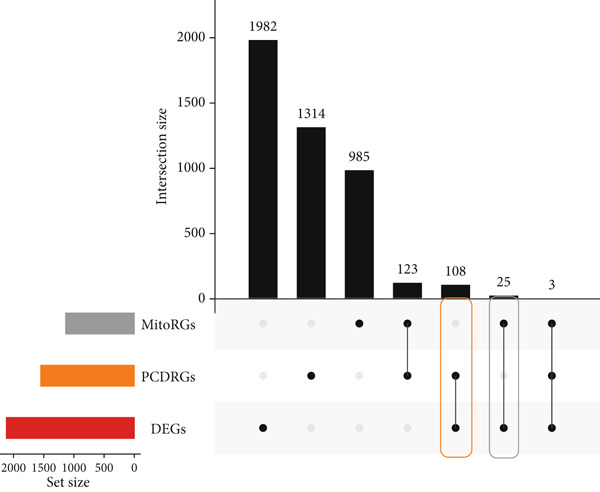
(d)

**Figure:**
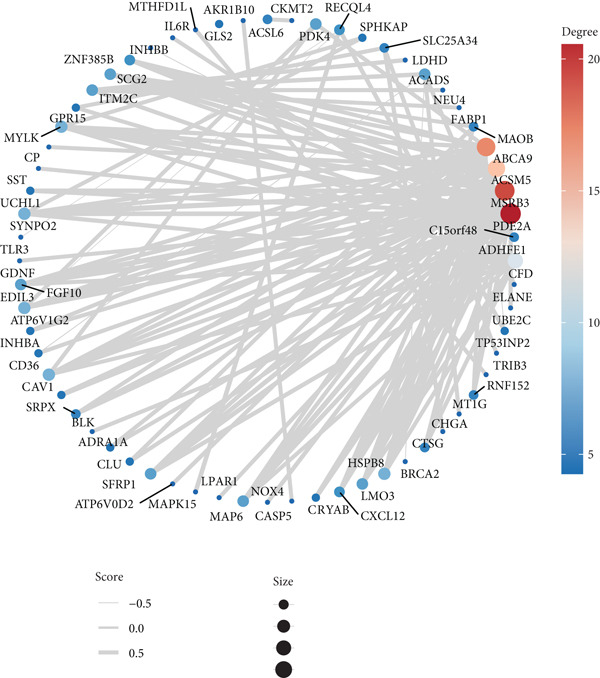
(e)

**Figure:**
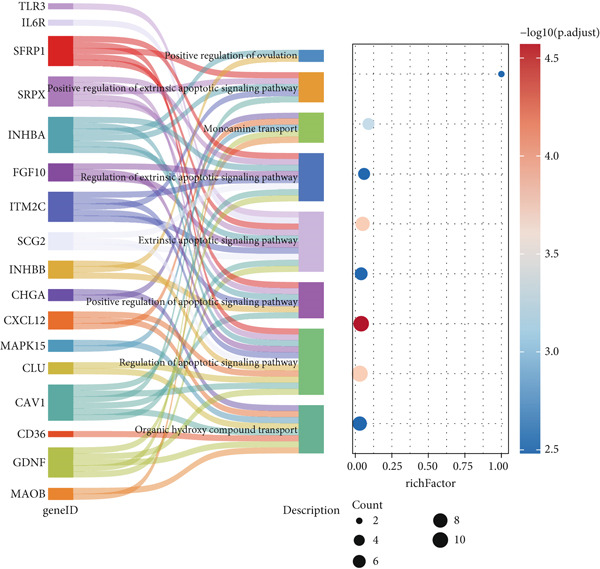
(f)

**Figure:**
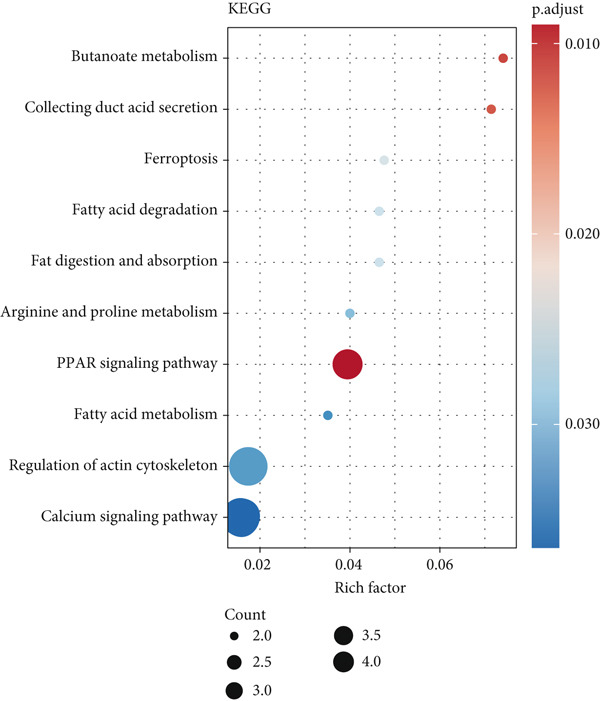
(g)

### 3.2. Identifying mtPCD‐Associated Molecular Subtypes

By increasing the clustering variable (*k*) from 2 to 5, the results showed that when *k* = 2, the intragroup associations were the highest, suggesting that the CRC samples could be well separated into two clusters according to the obtained 64 DEmtPCD coexpressed genes, including C1 and C2 (Figure 3a). PCA observed that the samples in the two subtypes could be well separated (Figure 3b), and patients in the C1 group had better survival outcomes (*p* = 0.046; Figure 3c). Also, the GSVA revealed that a total of 42 DE HALLMARK pathways between C1 and C2 subtypes were identified, containing 21 upregulated and 21 downregulated pathways (Figure 3d). Additionally, the fraction of a total of 14 immune cells showed a significant difference between C1 and C2 subtypes (both *p* < 0.05; Figure 3e), such as CD8 T cells, activated NK cells, and M2 macrophages, as well as immune score, tumor purity, and stroma score (both *p* < 0.001; Figure 3f). Also, the enrichment analysis uncovered that the C2 subtype was involved in focal adhesion, calcium signaling pathway, and ECM receptor interaction pathways (Figure 3g). In addition, a total of 2773 DEGs were screened between C1 and C2 subtypes (Figure S1A), and 33 common genes were obtained after intersecting the 64 DEmtPCD coexpressed genes and the 2773 DEGs (Figure S1B). Enrichment analysis found that these 33 genes were enriched in the cGMP‐PKG signaling pathway, the regulation of actin cytoskeleton, and the calcium signaling pathway (Figure S1C).

Figure 3Identifying mtPCD‐associated molecular subtypes. (a) CRC patients grouped into two clusters according to the consensus clustering matrix (*k* = 2). (b) PCA. (c) Survival analysis of patients in different subtypes. (d) GSVA employed to identify differentially expressed HALLMARK pathways between different subtypes. (e) Immune infiltration analysis.  ^∗^ *p* < 0.05,  ^∗∗^ *p* < 0.01, and  ^∗∗∗^ *p* < 0.001. (f) Difference in immune score, tumor purity, and stroma score between different subtypes.  ^∗∗∗^ *p* < 0.001. (g) Enrichment analysis of the different subtypes.

**Figure:**
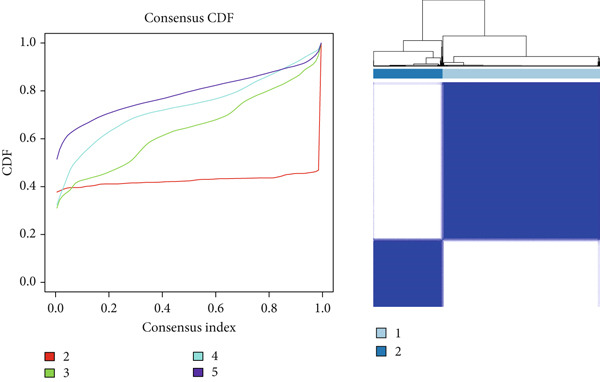
(a)

**Figure:**
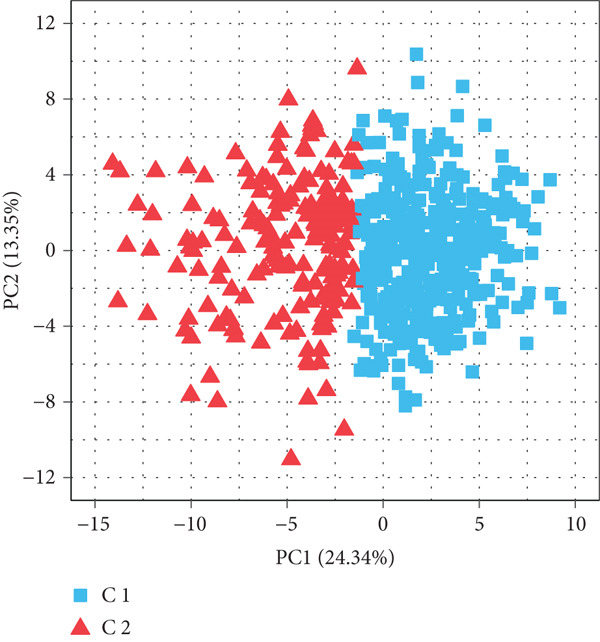
(b)

**Figure:**
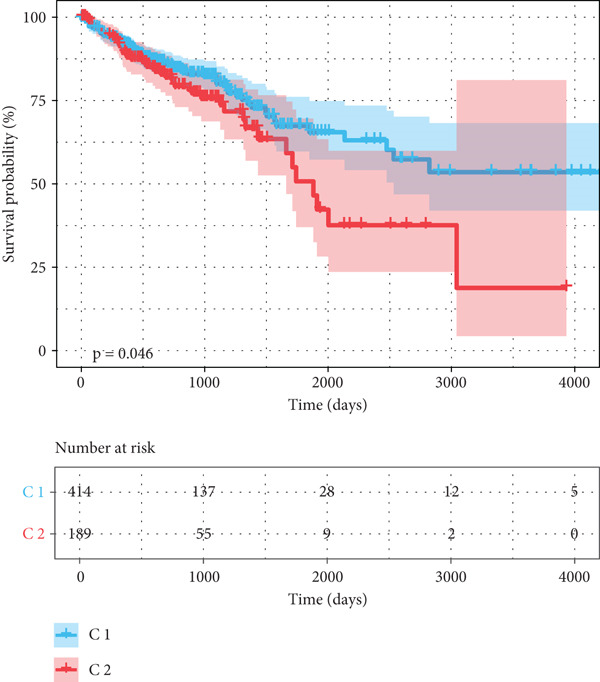
(c)

**Figure:**
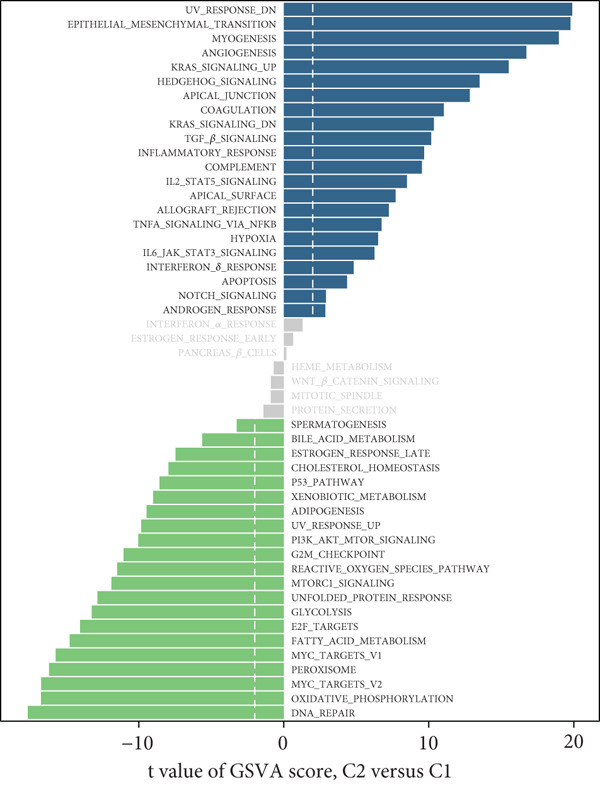
(d)

**Figure:**
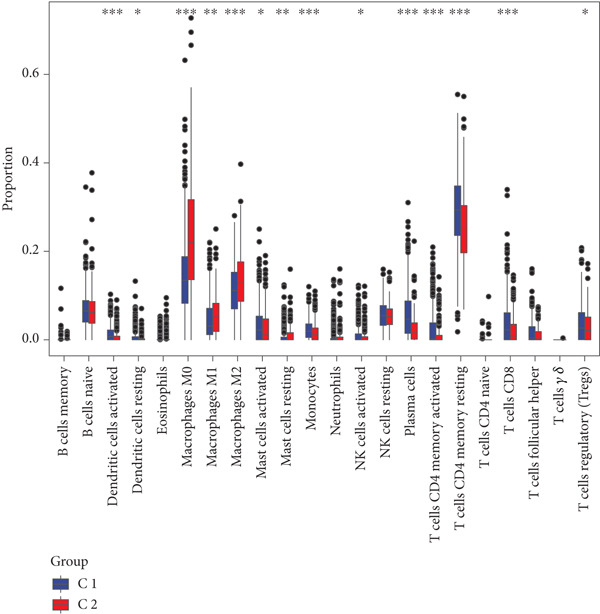
(e)

**Figure:**
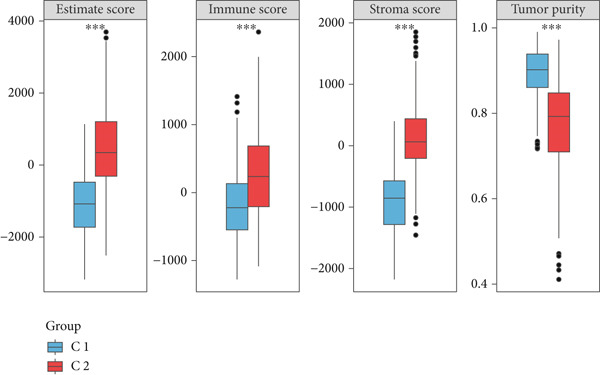
(f)

**Figure:**
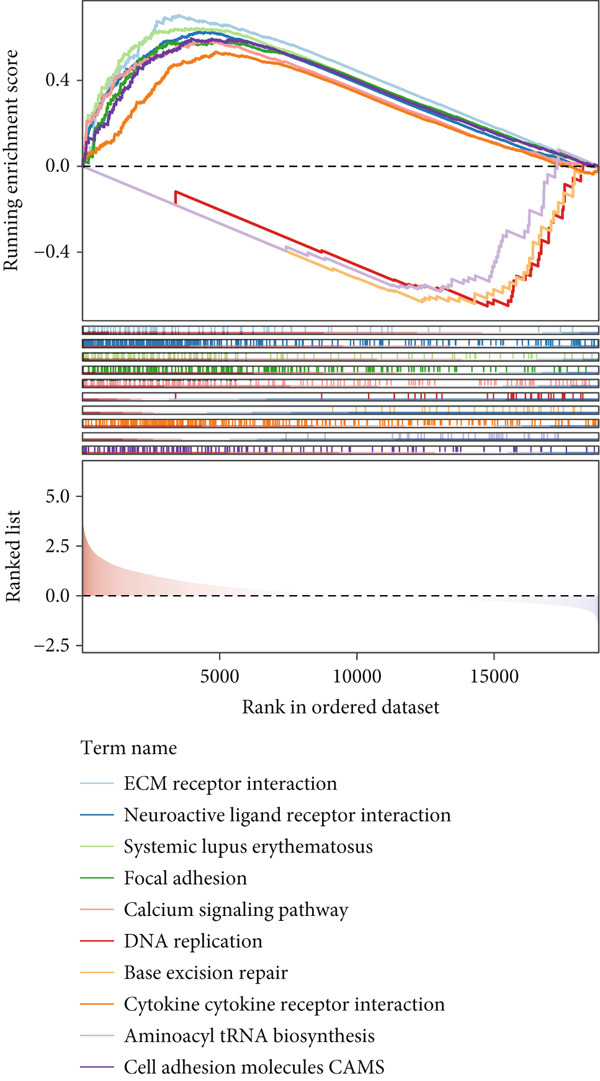
(g)

### 3.3. Prognostic Model Construction and Validation

After univariate Cox regression analysis, eight DEmtPCD coexpressed genes related to the prognosis of patients were screened (Figure 4a). Furthermore, LASSO analysis detected seven prognosis‐related DEmtPCD coexpressed genes with the best weighting coefficient (Figure 4b). Then, multivariate Cox regression analysis and the “step” function screened four key genes used for prognostic model construction, namely, ACSL6, INHBB, GPR15, and SRPX (Figure 4c). In the training set, patients in the H‐risk group died earlier and had a lower survival probability than those in the L‐risk group, and the expression levels of the four key genes were obviously different between the H‐ and L‐risk groups (Figure 4d,e). In addition, the survival time, risk score, OS, and expression of the four key genes in the validation set (GSE17537) were in line with those in the training set (Figure 4g,h). Also, the AUCs for OS at 1, 3, and 5 years were 0.667, 0.665, and 0.603 in the TCGA set, separately (Figure 4f), and 0.759, 0.766, and 0.662, separately, in the GSE17537 dataset (Figure 4i). Furthermore, clinical features pathologic T, pathologic N, and stage were closely associated with risk score (Figure 4j). Notably, in both subtypes and risk groups, the proportion of deaths was higher in the poor‐prognosis group (Figure 4k).

Figure 4Construction and validation of a prognostic model. (a) Univariate Cox regression analysis. (b) Least absolute shrinkage and selection operator (LASSO) coefficient profiles and coefficient profile plot produced against the log (lambda) sequence in the LASSO model. (c) Multivariate Cox regression analysis. Risk score distribution, survival status, and expression level in (d) The Cancer Genome Atlas (TCGA) set and (g) the GSE17537 dataset. Survival analysis in (e) the TCGA set and (h) the GSE17537 dataset. Receiver operating characteristic (ROC) curves in (f) the TCGA set and (i) the GSE17537 dataset. (j) Difference in clinical features between risk groups.  ^∗^ *p* < 0.05,  ^∗∗^ *p* < 0.01, and  ^∗∗∗^ *p* < 0.001. (k) Correlation between subtypes, risk groups, and survival status.

**Figure:**
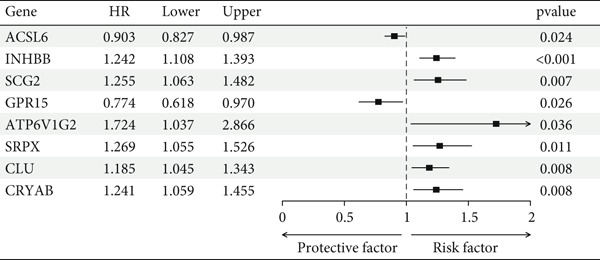
(a)

**Figure:**
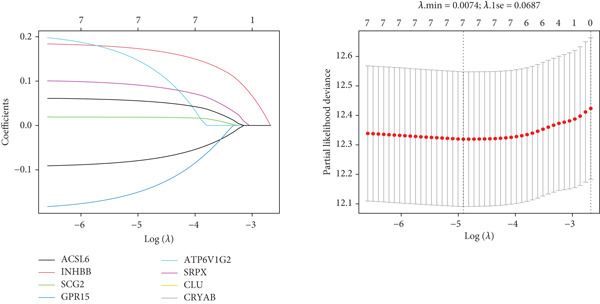
(b)

**Figure:**
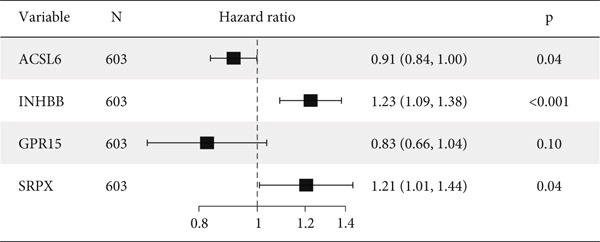
(c)

**Figure:**
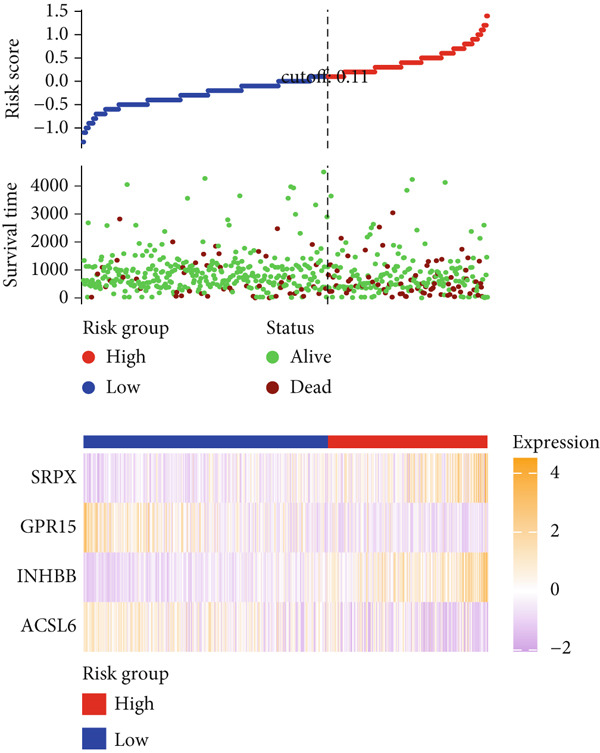
(d)

**Figure:**
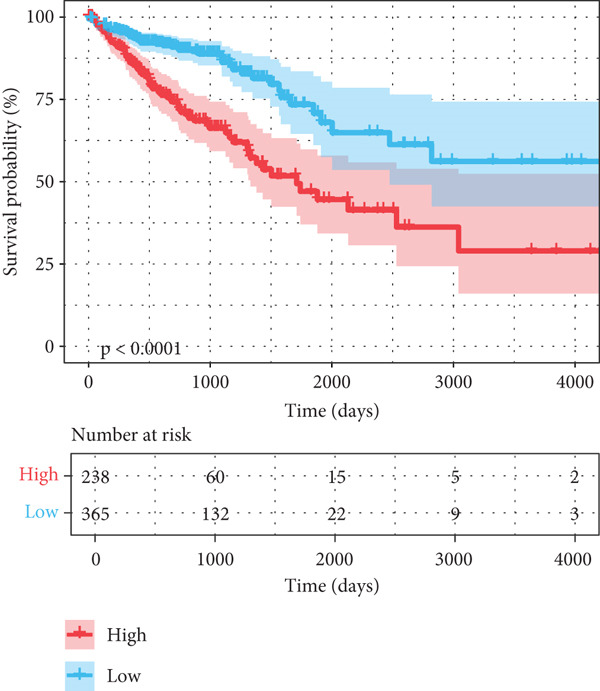
(e)

**Figure:**
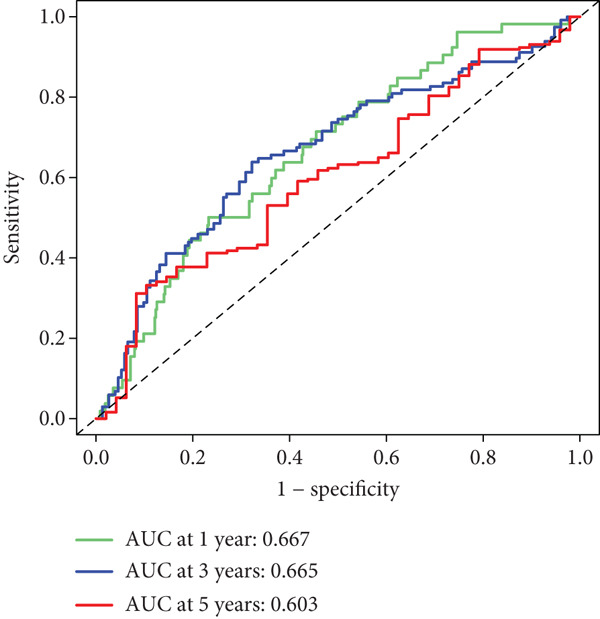
(f)

**Figure:**
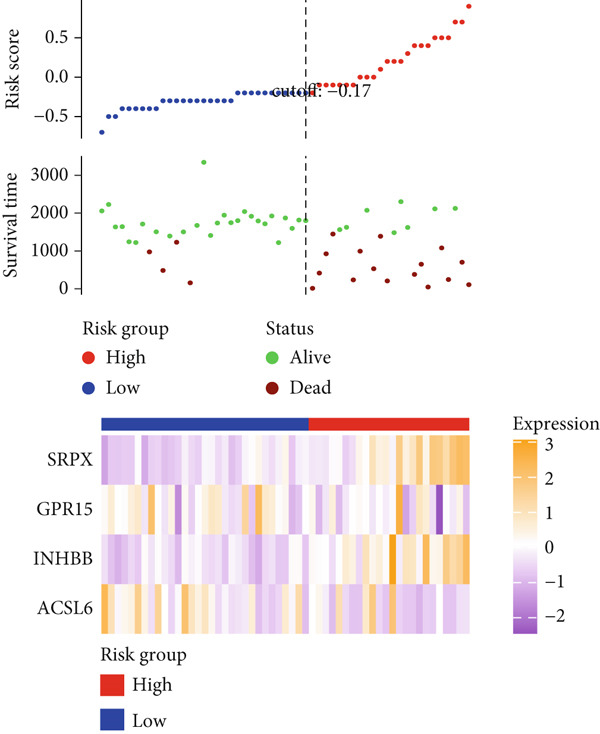
(g)

**Figure:**
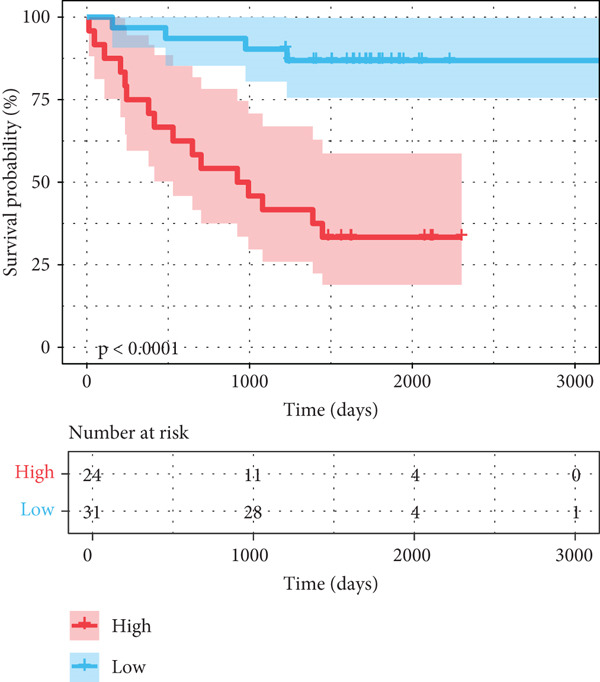
(h)

**Figure:**
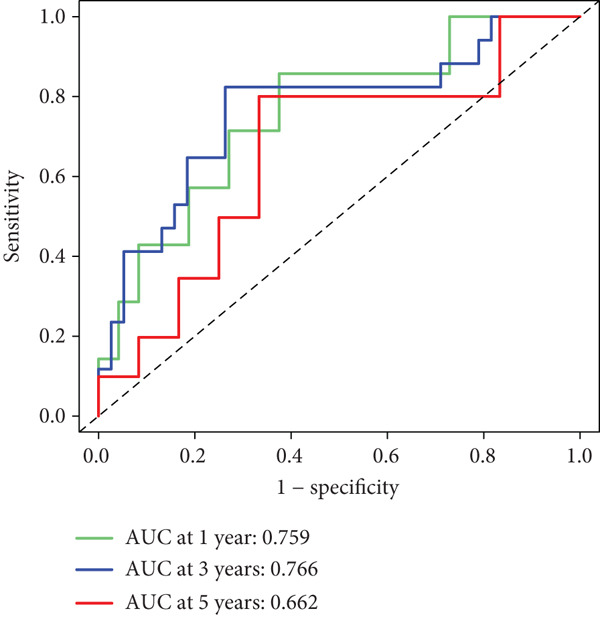
(i)

**Figure:**
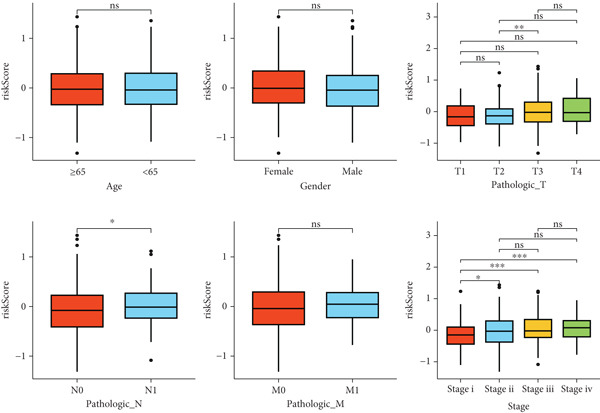
(j)

**Figure:**
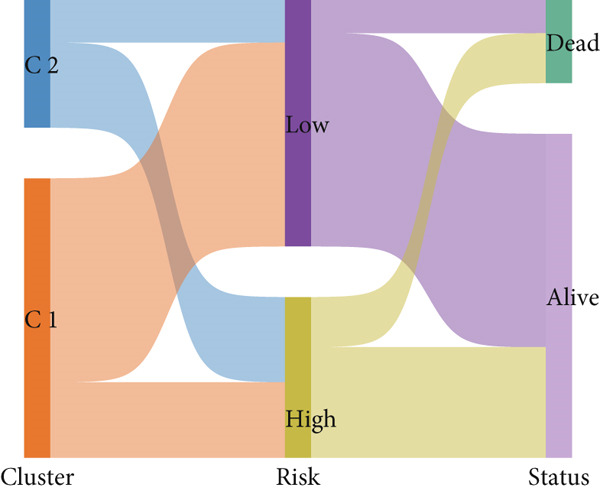
(k)

### 3.4. Correlation Analysis of Risk Score and Immunity

Correlation between immune microenvironments among risk groups was analyzed. Immune checkpoint analysis found that the expression of immune checkpoints between the L‐ and H‐risk groups showed a significant difference, containing PDCD1, PDCD1LG2, and CCL2 (both *p* < 0.05; Figure 5a). A total of nine immune cells revealed a significant difference between the L‐ and H‐risk groups (both *p* < 0.05; Figure 5b), such as neutrophils, activated NK cells, and activated dendritic cells (DCs), as well as estimated score and stroma score (both *p* < 0.001; Figure 5c). Furthermore, patients in the L‐risk group had lower TIDE scores (Figure 5d). Based on the TIDE score of targeted immune checkpoints, the sensitivity of the H‐risk group patients to immune checkpoints and immunotherapy was analyzed, and it was found that patients in the H‐risk group had weaker immune responses (Figure 5e). Besides, the top 20 mutated genes in the H‐ and L‐risk groups were separately detected, and the results found that APC was the most significant mutated gene in both the H‐ and L‐risk groups (Figure 5f,g). Also, the enrichment analysis indicated that numerous pathways were activated in the H‐risk group, such as ECM receptor interaction, focal adhesion, and pathways in cancer (Figure 5h).

Figure 5Correlation analysis of risk score and immunity. (a) Difference in the expression of immune checkpoints between the L‐ and H‐risk groups. (b) Difference in the proportion of immune cells between the L‐ and H‐risk groups. (c) Difference in the estimate score, immune score, and stroma score between the L‐ and H‐risk groups. (d) Difference in TIDE scores between the H‐ and L‐risk groups. (e) Difference in TIDE score targeted immune checkpoints between the L‐ and H‐risk groups. Top 20 mutated genes in the (f) H‐ and (g) L‐risk groups. (h) Enrichment analysis of the different risk groups.  ^∗^ *p* < 0.05,  ^∗∗^ *p* < 0.01, and  ^∗∗∗^ *p* < 0.001.

**Figure:**
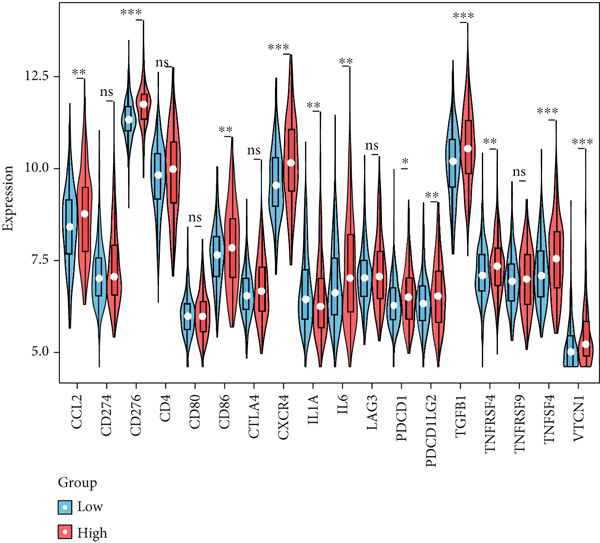
(a)

**Figure:**
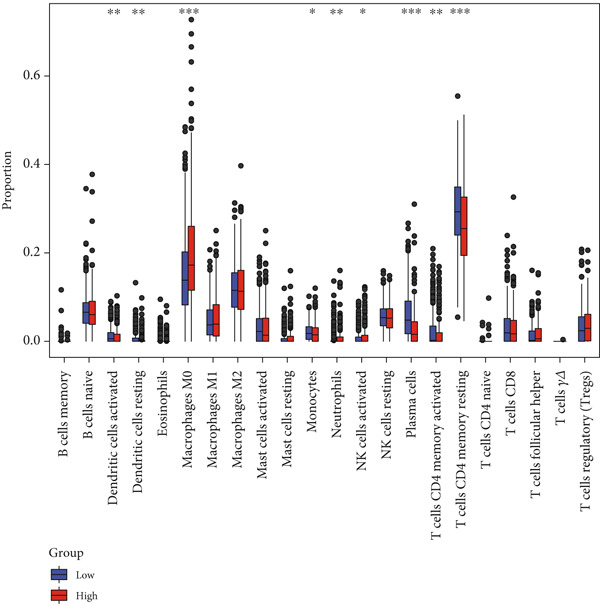
(b)

**Figure:**
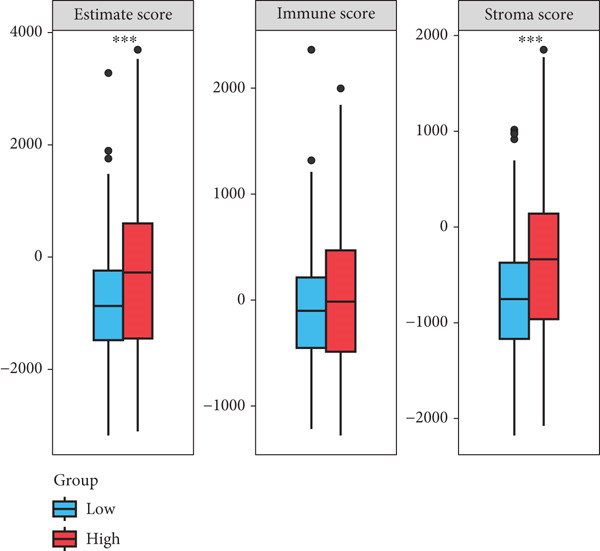
(c)

**Figure:**
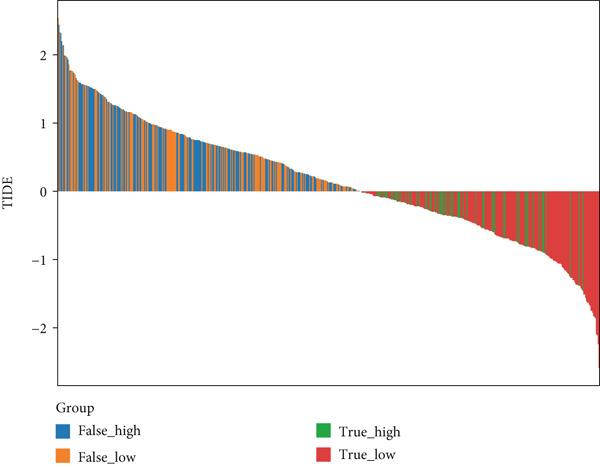
(d)

**Figure:**
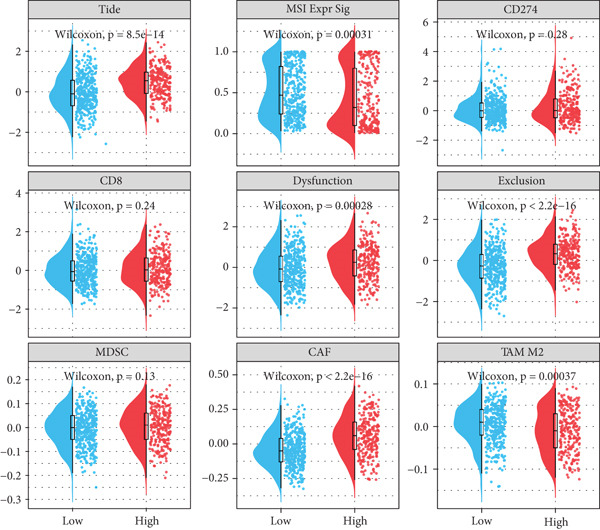
(e)

**Figure:**
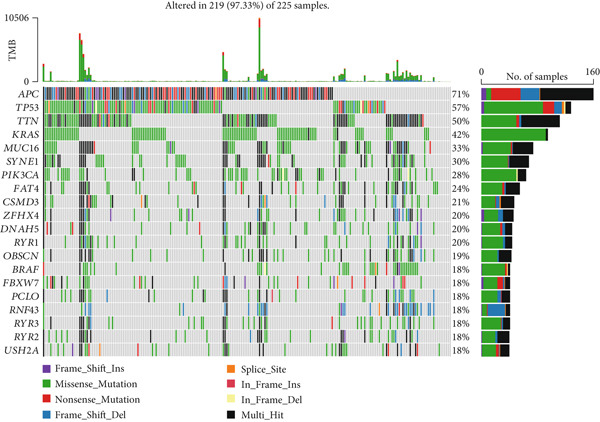
(f)

**Figure:**
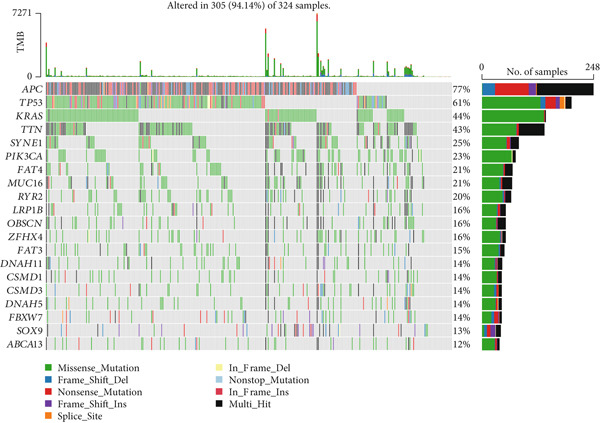
(g)

**Figure:**
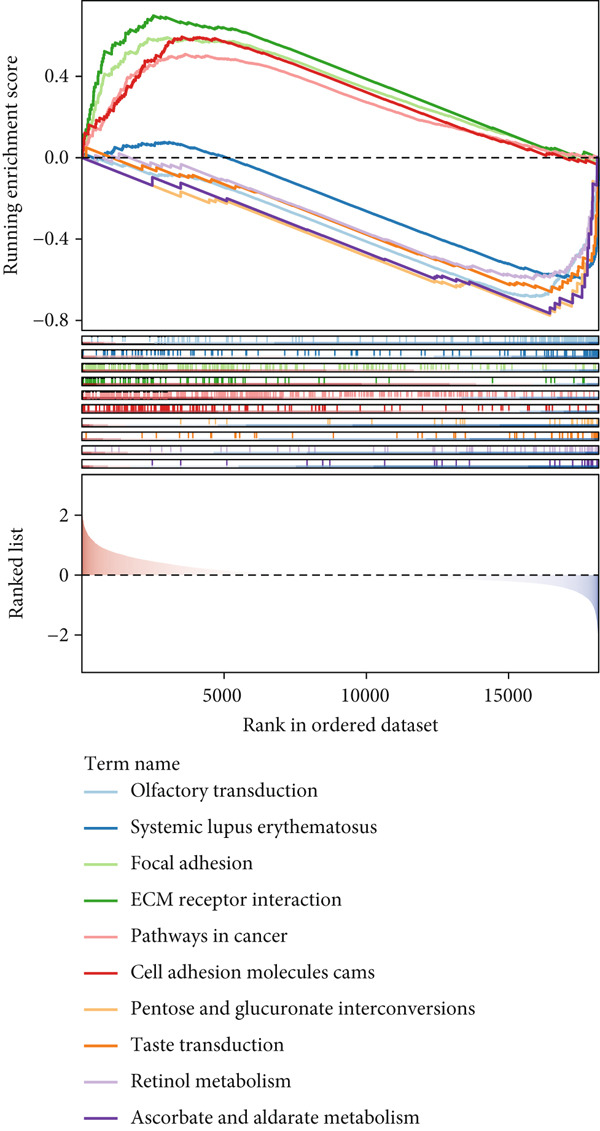
(h)

### 3.5. Drug Sensitivity and Key Gene Validation

Our analysis revealed that IC_50_ values of some chemotherapeutic agents showed a significant difference between the L‐ and H‐risk groups, containing AZD1332‐1463, IGF1R‐3801‐1738, and XAV939‐1268 (Figure 6a). In addition, correlation analysis found that Vinblastine, Obatoclax mesylate, and Temsirolimus were significantly positively correlated with ACSL6 and INHBB, while Docetaxel, 17‐AAG, and Bleomycin (50 *μ*M) were significantly negatively correlated with SRPX (Figure 6b). Besides, the expression of four key genes was validated in the GSE87211 dataset, and the results were in line with the expression in the TCGA set (Figure 6c,d). Also, these four key genes were validated and could be used as prognostic genes in CRC (Figure 6e).

Figure 6Drug sensitivity and key genes validation. (a) Differences in chemotherapeutic agents showed a significant difference between the L‐ and H‐risk groups. (b) Correlation between four key genes and chemotherapeutic agents. The expression of four key genes in (c) the TCGA set and (d) the GSE87211 dataset.  ^∗∗∗^ *p* < 0.001. (e) Survival analysis of four key genes in the GSE87211 dataset.

**Figure:**
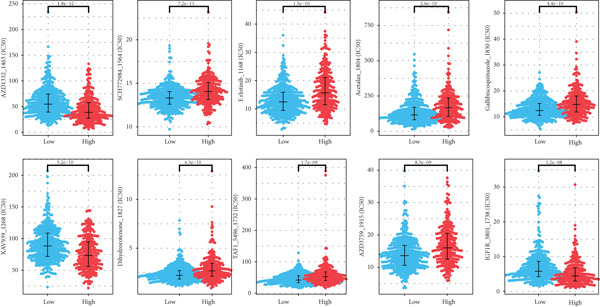
(a)

**Figure:**
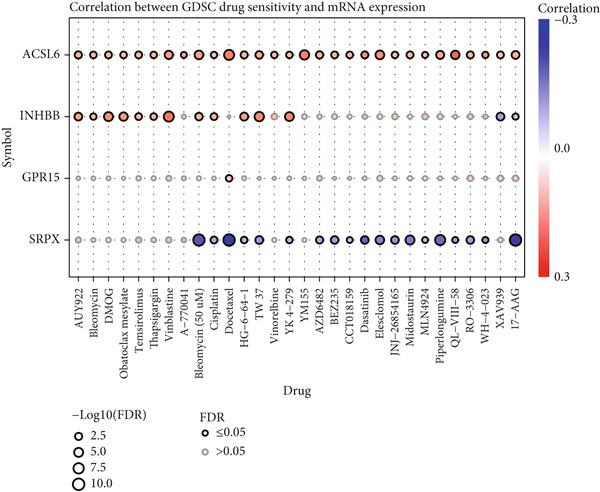
(b)

**Figure:**
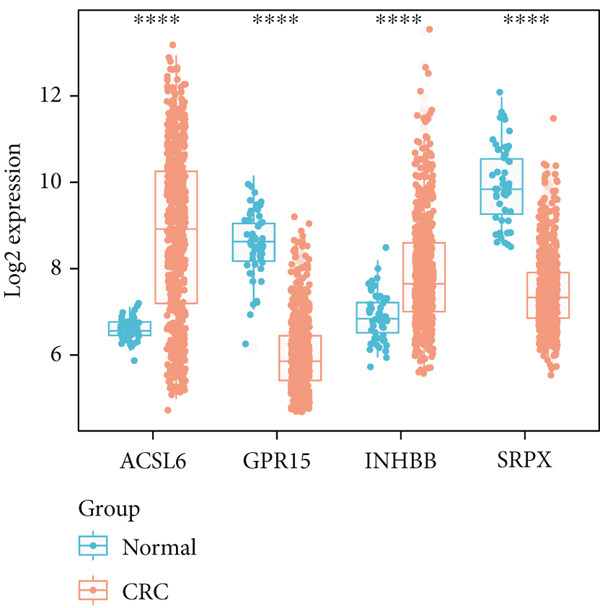
(c)

**Figure:**
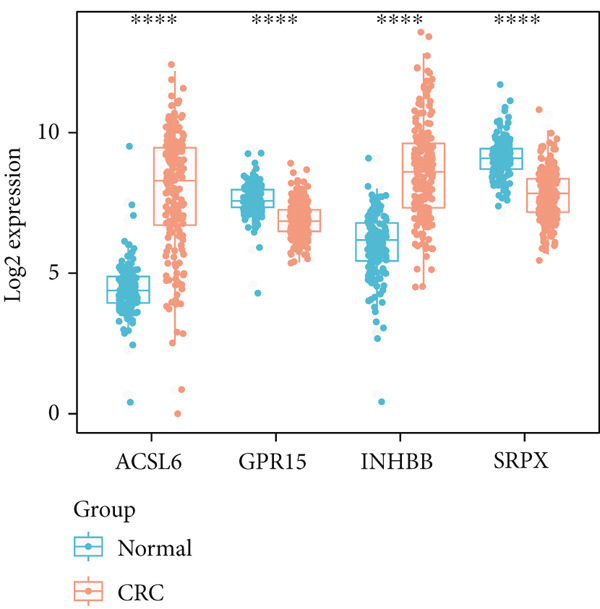
(d)

**Figure:**
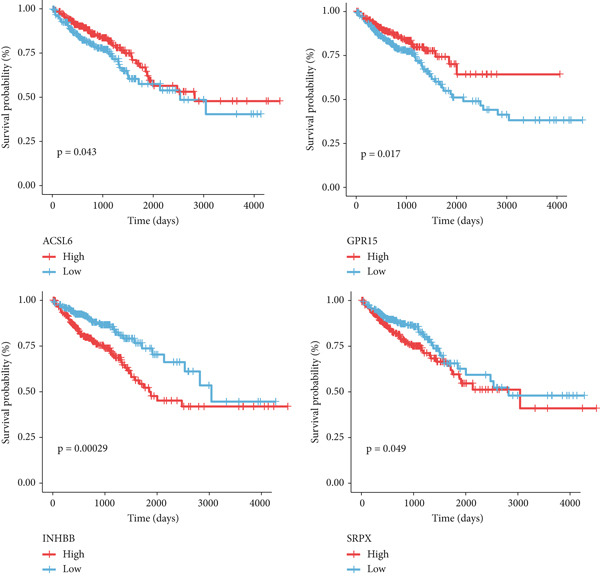
(e)

### 3.6. INHBB Accelerates the Proliferation and Migration of CRC Cells

To further validate the four key genes, the qRT‐PCR and western blot were performed. The results showed that the expression levels of ACSL6, GPR15, and INHBB were both obviously elevated in CRC cells compared to those in NCM460 cells, while SRPX was decreased (both *p* < 0.01; Figure 7a,b). Due to the expression of GPR15 in CRC cells being inconsistent with the bioinformatics analysis results (Figure 6c,d), the expression of ACSL6 and the survival analysis results being contradictory (Figures 6c, 6d, and 6e), and the function of SRPX in CRC reported [31–33], thus, the influence of INHBB in CRC was explored in vitro. Also, the expression level of INHBB was highest in SW620 cells; thus, the SW620 cell was chosen for subsequent analysis. Considering the upregulation of INHBB in CRC, sh‐INHBB lentivirus was used to explore, and the transfection efficiency was measured (Figure 8a,b). Notably, the downregulation of INHBB effectively alleviated the proliferation and migration of CRC cells (both *p* < 0.01; Figures 8c, 8d, and 8e). These data confirm that INHBB accelerates the proliferation and migration of CRC cells.

Figure 7Four key genes validation in vitro. (a) The mRNA expression levels of ACSL6, GPR15, INHBB, and SRPX detected by qRT‐PCR. (b) The protein expression levels of ACSL6, GPR15, INHBB, and SRPX detected by western blot.  ^∗^ *p* < 0.05 and  ^∗∗^ *p* < 0.01.

**Figure:**
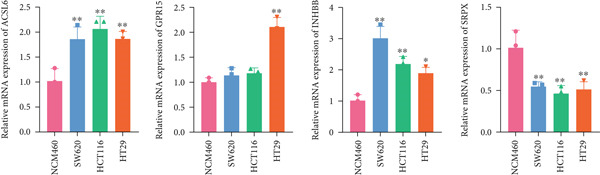
(a)

**Figure:**
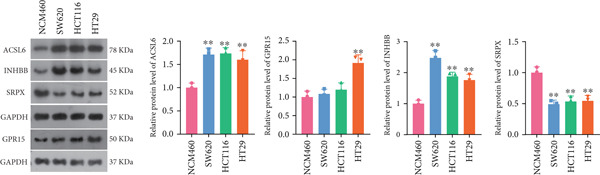
(b)

Figure 8INHBB accelerates the proliferation and migration of CRC cells. Transfection efficiency measured in SW620 cells by (a) qRT‐PCR and (b) western blot. (c) Cell proliferation detected by CCK‐8. (d) Cell migration detected by the Scratch wound healing assay. Scale bar = 50 * μ*m. (e) Cell proliferation detected by colony formation assay.  ^∗∗^ *p* < 0.01 compared with the control group, ^##^ *p* < 0.01 compared with the sh‐NC group.

**Figure:**
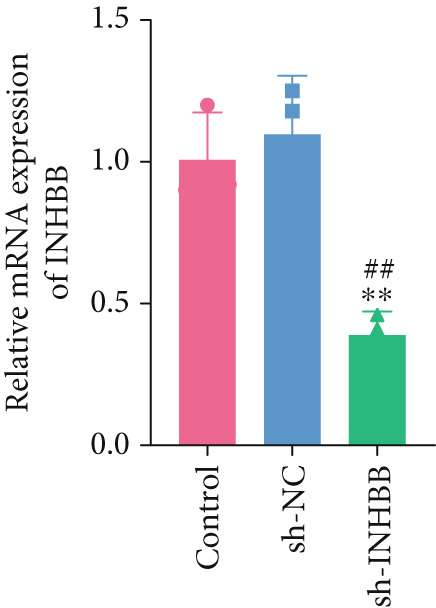
(a)

**Figure:**
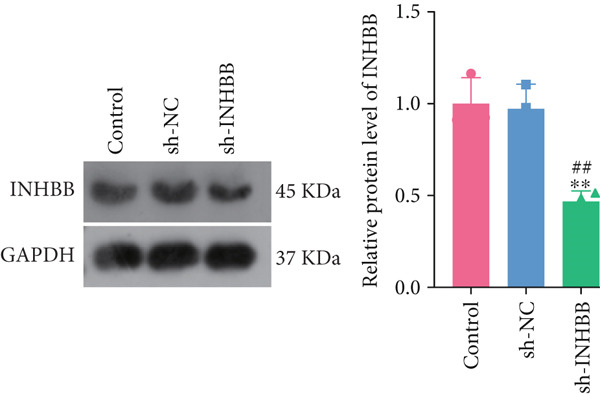
(b)

**Figure:**
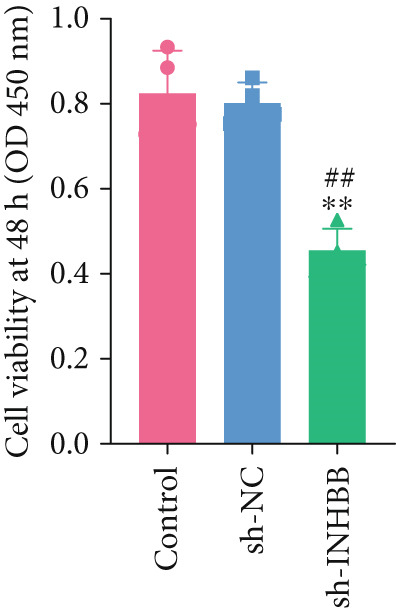
(c)

**Figure:**
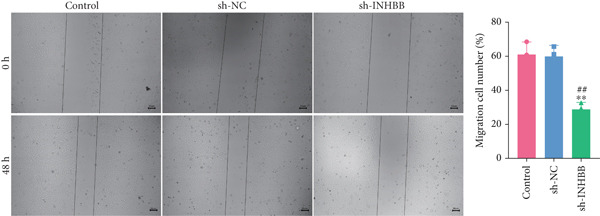
(d)

**Figure:**
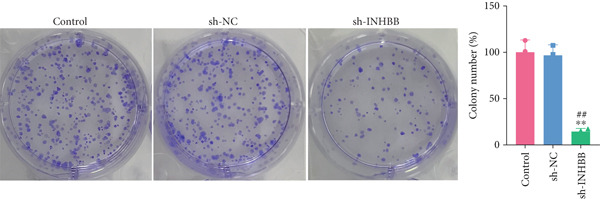
(e)

## 4. Discussion

Currently, CRC has become one of the most important challenges facing health systems in the world [34, 35]. Our study constructed a novel mtPCDRG prognostic model that could predict the survival outcome of patients with CRC based on four key genes, namely, ACSL6, INHBB, GPR15, and SRPX. In addition, inhibition of INHBB attenuated the proliferation and migration of CRC cells.

Long‐chain acyl‐coenzyme A synthases (ACSLs) are responsible for catalyzing fatty acids into the corresponding fatty acyl‐CoAs [36]. Teodoro et al. revealed that ACSL6 gene inhibition in rat primary myotubes decreased lipid accumulation, as well as activated the higher mitochondrial oxidative capacity program and fatty acid oxidation through the AMPK/PGC1‐alpha pathway [37]. Currently, accumulating evidence suggests that the expression of ACSLs is dysregulated in cancer patients. Di et al. found that ACSL6 is upregulated in liver cancer and associated with poor survival outcomes of patients [38]. However, Hua et al. also revealed that when compared to adjacent normal tissues, the expression of ACSL6 is remarkably elevated in triple‐negative breast cancer tissues, while high expression of ACSL6 was closely associated with a favorable prognosis of triple‐negative breast cancer patients [39]. Similarly, in this study, we also found that the ACSL6 expression was obviously increased in CRC samples compared to that in the control, and high expression of ACSL6 is correlated with favorable survival outcomes in CRC patients. Subsequent studies should be conducted to confirm this result. GPR15 is a widely studied orphan G protein–coupled receptor, and Adamczyk et al. uncovered that GPR15 promotes regulatory T‐cell recruitment, thereby facilitating CRC tumorigenesis [40]. Also, GPR15, as a binding partner of TME5, and TME5 rescued growth inhibition and apoptosis caused by calcineurin inhibitor FK506 in vascular ECs isolated from wild‐type C57BL/6 mice [41]. In addition, SRPX has been reported to participate in the pathogenesis of cancers. For instance, Ampudia‐Mesias et al. suggested that SRPX expression was related to the tumor grade of glioblastoma [42]. Liu et al. indicated that SRPX accelerates the invasiveness of ovarian carcinoma through mediating cancer‐associated fibroblasts [43]. Also, Pan et al. highlighted the pivotal roles of PCDRG SRPX in the molecular subtyping and prognostic forecasting of uterine corpus endometrial carcinoma [44]. Furthermore, INHBB encodes the Inhibin Subunit Beta B, which participates in the formation of the TGF‐*β* family members [45, 46]. Shi et al. have reported that TGF‐*β*/JNK axis mediates mitochondrial damage and macrophage cGAS‐STING activation in liver Mallory–Denk body pathogenesis [47]. Sun et al. have illustrated that TGF‐*β*1 attenuates mitochondrial bioenergetics in pulmonary arterial endothelial cells via the disruption of carnitine homeostasis [48]. Yang et al. have uncovered that Stanniocalcin‐1 suppresses TGF‐*β*‐induced mitochondrial dysfunction and cellular fibrosis in human renal proximal tubular cells [49]. Also, Zhou et al. unveiled that PCDRG INHBB is involved in the development of osteoarthritis synovitis [50]. In addition, a previous study illustrated that INHBB is mediated by methylation and strongly associated with the metastasis of CRC [51]. Yang et al. elucidated that INHBB activated EGFR signaling to facilitate tumor stemness and aggressiveness of glioblastoma [52]. This study also validated the four key genes using qRT‐PCR and western blot, and the results showed that the expression levels of ACSL6, GPR15, and INHBB were both obviously elevated in CRC cells compared to those in NCM460 cells, while SRPX was decreased. Except for the GPT results, all other results are consistent with the bioinformatics analysis results. Notably, the downregulation of INHBB effectively alleviated the tumorigenesis of CRC cells. These data confirm that INHBB accelerates the tumorigenesis of CRC cells, which provides novel biomarkers for CRC screening.

The TME exerts a significant function in the pathogenesis of tumors [53, 54]. Herein, the proportion of immune cells between the L‐ and H‐risk groups was calculated. The “CIBERSORT” algorithm revealed that a total of nine immune cells revealed a significant difference between the L‐ and H‐risk groups, such as neutrophils, activated NK cells, and activated DCs. Neutrophils are the most abundant myeloid cells in human blood with cancer‐promoting effects [55]. NK cells are innate lymphocytes that are capable of recognizing and killing cancer cells [56]. DCs are a group of innate immune cells that present heterogeneous antigens and mediate adaptive immunity, containing anticancer [57]. These data suggested that neutrophils, activated NK cells, and activated DCs might exert a significant function in the pathogenesis of CRC.

Tumor cells with a lower TIDE score are less likely to have immune escape, which means a higher response rate to immune checkpoint inhibitor therapy [58]. This study found that patients in the L‐risk group had lower TIDE scores, suggesting that the patients in the L‐risk group had better immunotherapeutic potential. Also, the IC_50_ value of some chemotherapeutic agents was lower in the H‐risk group, containing AZD1332‐1463, IGF1R‐3801‐1738, and XAV939‐1268, indicating that AZD1332‐1463, IGF1R‐3801‐1738, and XAV939‐1268 were potentially beneficial for CRC patients in the H‐risk group. Huang and He revealed that AZD1332‐1463 was sensitive to patients with high 7‐methylguanosine‐related prognostic signature risk scores [59]. In addition, correlation analysis found that Vinblastine, Obatoclax mesylate, and Temsirolimus were both significantly positively correlated with ACSL6 and INHBB, while Docetaxel, 17‐AAG, and Bleomycin (50 *μ*M) were significantly negatively associated with SRPX. Thus, our results may offer implications for subsequent drug research and immunotherapy for CRC patients.

Nevertheless, some limitations need to be considered. Firstly, the molecular mechanism of INHBB involved in CRC should be further explored. Secondly, further research is needed on the role of ACSL6, GPR15, and SRPX in CRC. Thirdly, the screened immune cells and chemotherapeutic agents should be further confirmed through experimental analyses. In addition, some assays on apoptosis or other cell death pathways should also be conducted. Lastly, the predicted performance of the constructed prognostic model should be tested at the clinical level.

## 5. Conclusion

In summary, this study constructed a novel mtPCD‐related prognostic model that could predict the prognosis of patients with CRC based on four key genes, namely, ACSL6, INHBB, GPR15, and SRPX. In addition, inhibition of INHBB attenuated the tumorigenesis of CRC cells. This study offers a theoretical foundation for drug research and immunotherapy for CRC patients.

## Disclosure

All authors read and approved the final manuscript.

## Conflicts of Interest

The authors have no relevant financial or non‐financial interests to disclose.

## Author Contributions

Yanqing Sun: conception and design of the research, acquisition of data, analysis and interpretation of data, and drafting the manuscript. Chun Gao: acquisition of data and analysis and interpretation of data. Dong Jia: statistical analysis. Qiang Ma: data curation and writing (review and editing). Wei Wang: conception and design of the research and revision of the manuscript for important intellectual content. Yanqing Sun and Chun Gao are co‐first authors and contributed equally to this work.

## Funding

This study was funded by the Key Research and Development Program of Gansu Province, No. 24YFFA069.

## General Statement

*Declaration of originality*. The authors confirm that this manuscript is their original work, has not been plagiarized, and all external content is appropriately referenced.

## Supporting Information

Additional supporting information can be found online in the Supporting Information section.

## Supporting information


**Supporting Information 1** Table S1: Identification of DEGs between CRC and normal samples.

**Supporting Information 2** Figure S1: DEGs screened between C1 and C2 subtypes. (A) Volcano plot of DEGs screened between C1 and C2 subtypes. (B) Venn diagram of 33 common genes. (C) Enrichment analysis of the 33 common genes.

## Data Availability

The datasets generated and analyzed during the current study are available from the corresponding author on reasonable request.
