# Supplementary figures and images for: A Novel Mitochondria‐Associated Programmed Cell Death–Related Prognostic Model and Validation of Oncogene INHBB in Colorectal Cancer
Source: Int J Genomics. 2025 Nov 26;2025:8691810. doi: 10.1155/ijog/8691810 (PMC12648302; doi:10.1155/ijog/8691810)

A

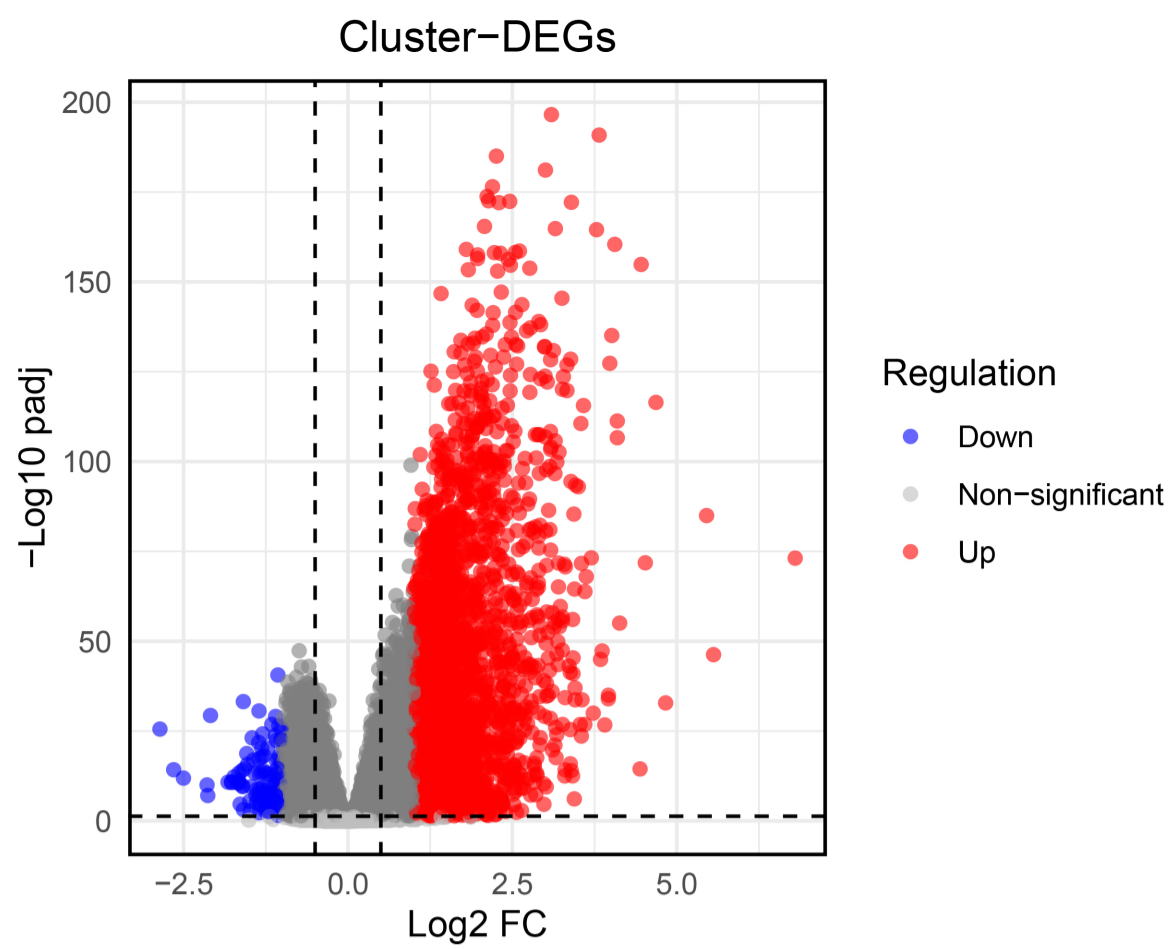

B

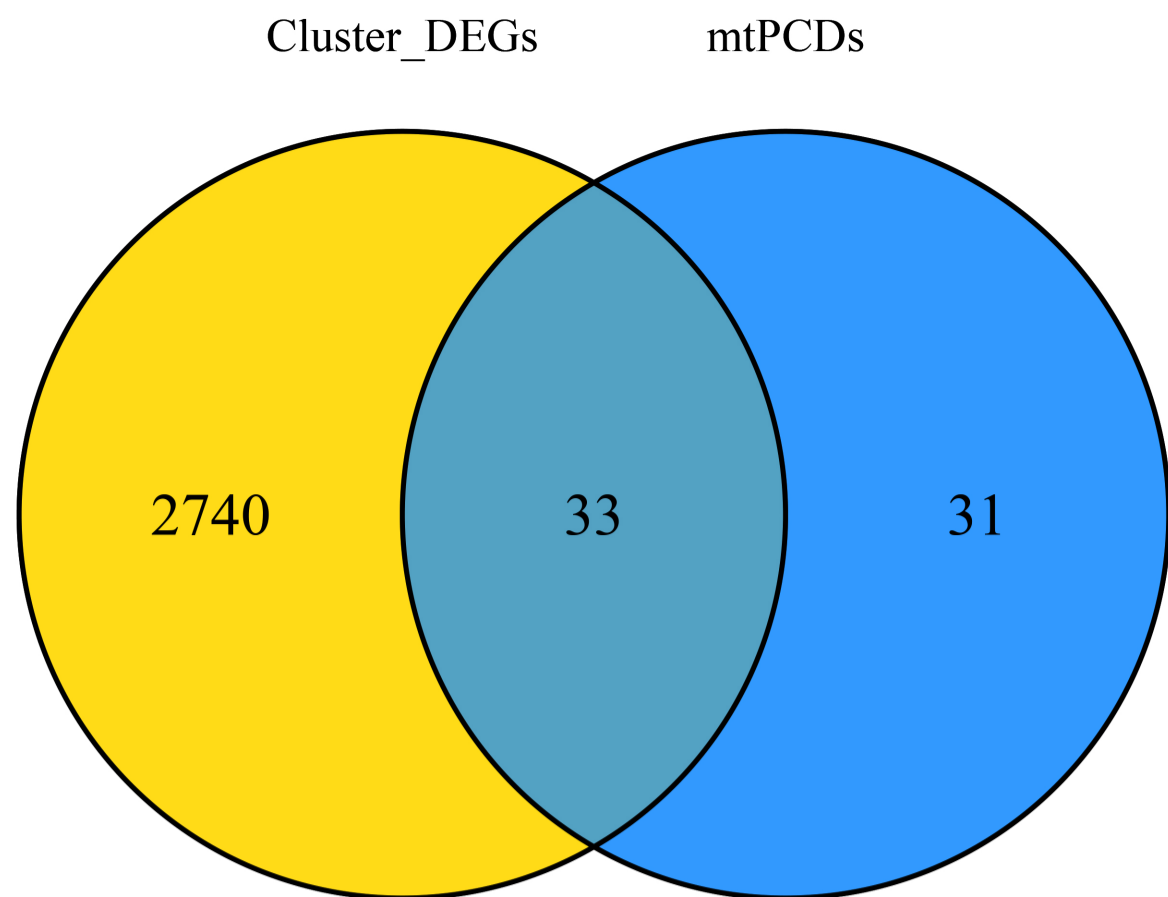

C

### GO Enrichment (Top 5)

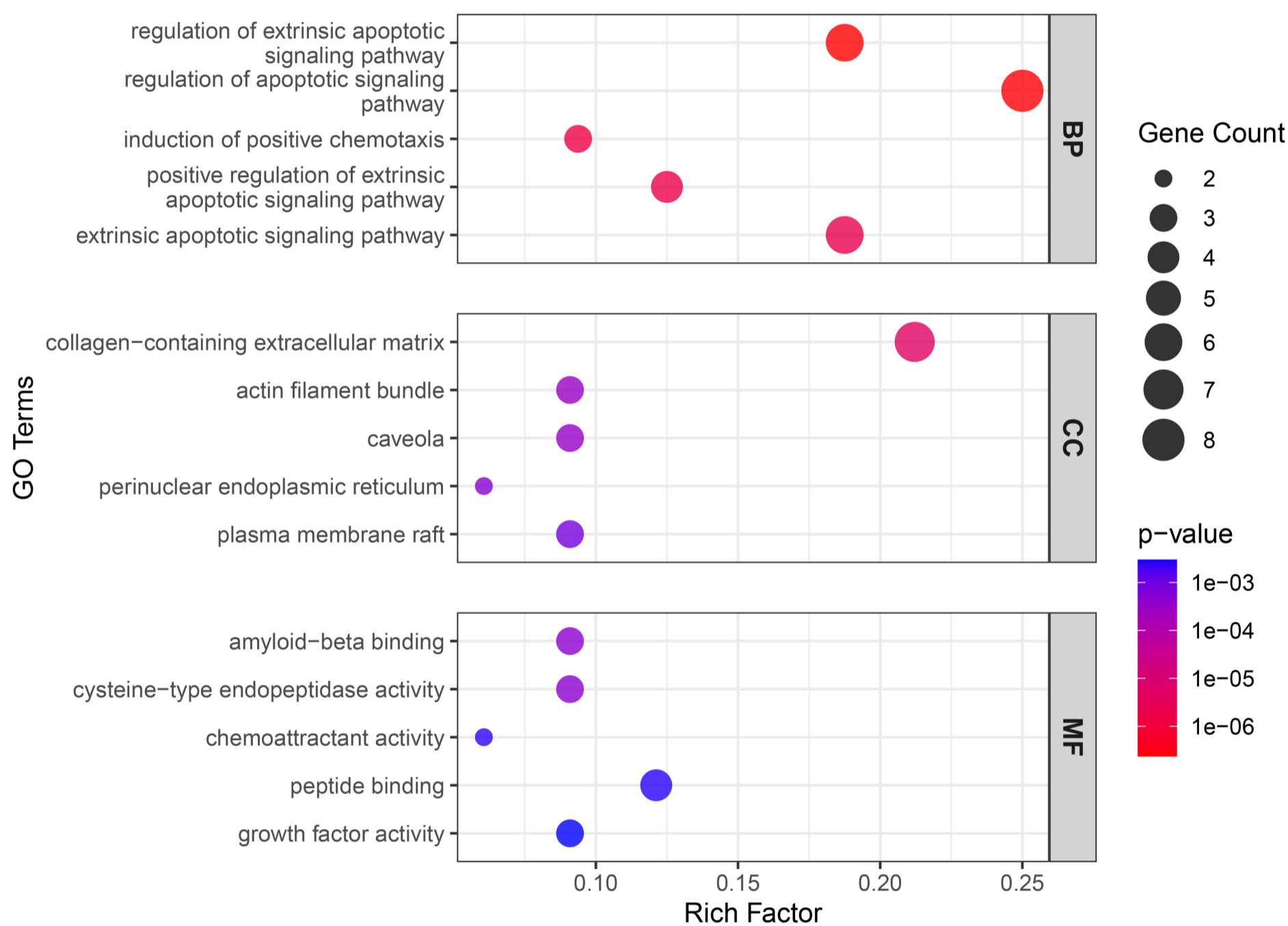

### KEGG Pathway Enrichment

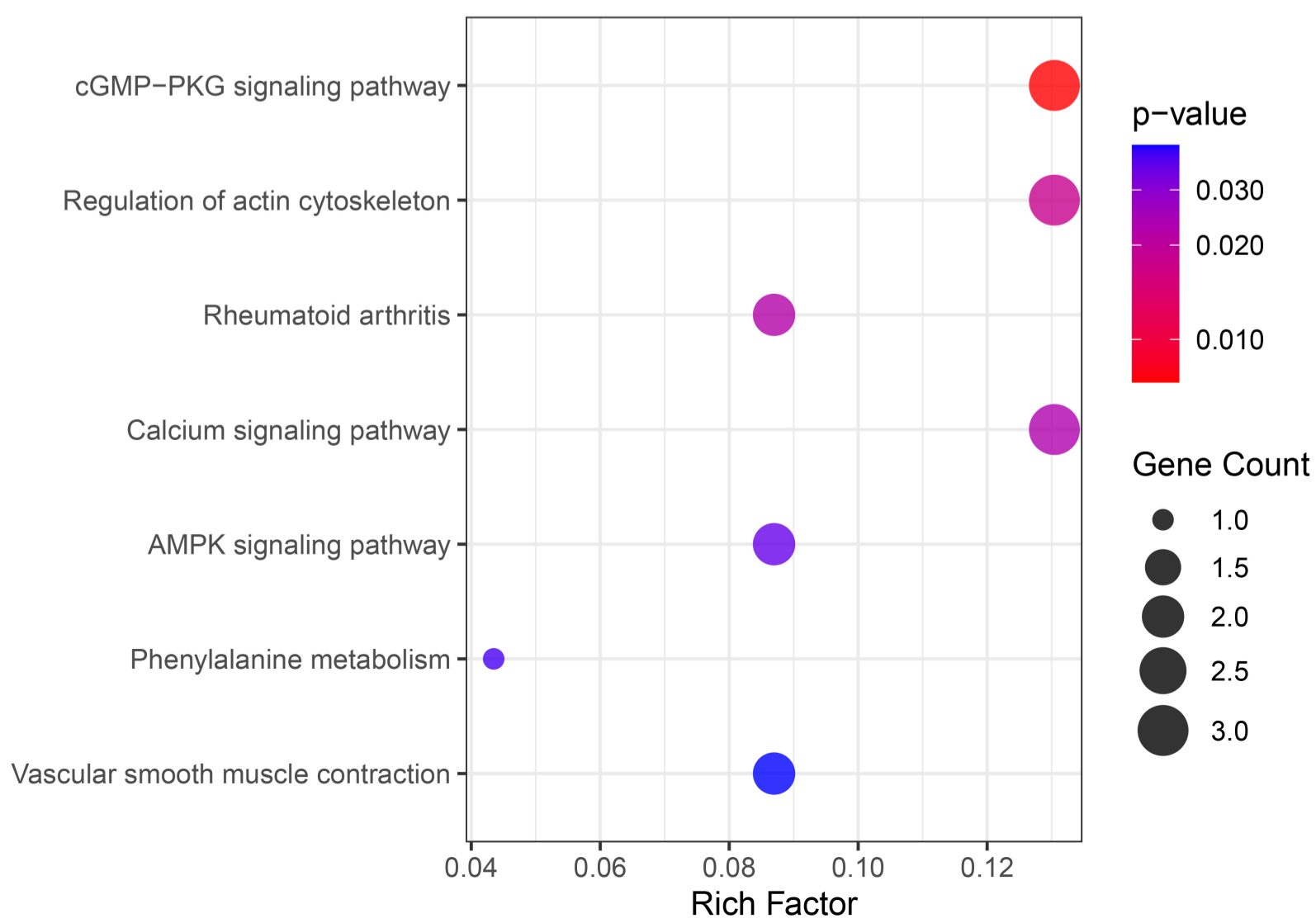

Supplement: Supplementary file 2 — Supporting Information 2 Figure S1: DEGs screened between C1 and C2 subtypes. (A) Volcano plot of DEGs screened between C1 and C2 subtypes. (B) Venn diagram of 33 common genes. (C) Enrichment analysis of the 33 common genes. [file IJOG-2025-8691810-s001.pdf]
